# Crack Growth Monitoring with Structure-Bonded Thin and Flexible Coils

**DOI:** 10.3390/s22249958

**Published:** 2022-12-17

**Authors:** Catalin Mandache, Richard Desnoyers, Yan Bombardier

**Affiliations:** Aerospace Research Centre, National Research Council Canada, 1200 Montreal Rd., Ottawa, ON K1A 0R6, Canada

**Keywords:** eddy current, printed circuit board coil, structural health monitoring

## Abstract

Structural health monitoring with thin and flexible eddy-current coils is proposed for in situ detection and monitoring of fatigue cracks in metallic aircraft structures, providing a promising means of crack sizing. This approach is seen as an efficient replacement to periodic inspections, as it brings economic and safety benefits. As such, printed-circuit-board eddy-current coils are viable for in situ crack monitoring for multi-layer, electrically conductive structures. They are minimally invasive and could be attached to or embedded into the evaluated structure. This work focuses on the monitoring of fatigue crack growth from a fastener hole with structure-bonded, thin, and flexible spiral coils. Numerical simulations were used for optimization of the driving frequency and selection of crack-sensitive coil parameters. The article also demonstrates the fatigue crack detection capabilities using spiral coils attached to a 7075-T6 aluminum coupon.

## 1. Introduction

Eddy-current testing is one of the mainstream non-destructive testing (NDT) techniques. It sees extensive use in the evaluation of electrically conductive materials for crack and corrosion detection [1], part and coating thickness [2], conductivity checks [3], proximity estimation [4], etc. Traditionally, its main applications are in the aerospace and power generation industries, and it is currently making its way into metal-based additive manufacturing. Although extensively used for the inspection of metallic aircraft structures and components, eddy-current testing suffers from limited interrogating depth, being essentially known as a surface and near-surface inspection technique. This shortcoming is evident for inspections of multi-layer structures, where structure disassembly may be required to allow access to the area of interest. However, nowadays, with the advancements in printed electronics and wireless communication, eddy-current techniques are resurging as an in situ means of NDT, i.e., in the form of structural health monitoring (SHM) for damage detection [5,6,7,8,9].

Printed-electronics technologies offer the advantage of short-series manufacturing, allowing for effective designs and application-specific optimizations. Printed circuit board (PCB) eddy-current coils on flexible substrates present the perfect embodiment of condition monitoring technologies [10,11]. They are minimally invasive and suitable for permanent attachment to or embedment into metallic structures. Reduction of inspection costs, along with increased safety with permanently mounted eddy-current sensors, were proposed more than two decades ago [12], but their capabilities and potential have not been yet fully harnessed. The use of surface-bonded eddy-current coils do not need on-board instrumentation, as they could be connected to auxiliary instrumentation only at the time of testing. Moreover, such sensors could be used to provide not only the crack size, but also the crack growth rate and assist in future structural material and design changes.

The advantages of structurally attached, embedded sensors for condition assessment are pushing the boundaries of on-demand maintenance. This approach is perceived to be more effective, faster, and cheaper than periodic inspections. While there is room for improvement in terms of sensitivity to damage quantification, at the present time, embedded sensors could be deployed for screening purposes, so as to prompt more involved NDT techniques. There are, however, doubts regarding the durability, survivability, and reliability of the in situ monitoring sensors and transducers [13]. A shortcoming of using structurally embedded or attached sensors is that, in most cases, and especially with eddy-current ones, the part evaluation is local, from a single or limited number of geometrical positions. This constraint makes the condition-monitoring approach have more of a screening role. In some cases, the sensor had a sacrificial role, as it becomes damaged when the crack crosses its surface area [14,15]. Local strain fields, detrimental to sensors, introduced by fatigue cracking, were measured with strain gauges and used to predict crack initiation and propagation [16].

Although eddy-current techniques are capable of detecting sub-surface discontinuities, the depth of the inspection area is limited to the near-surface by its governing physics principles. To overcome accessibility limitations to the areas to be monitored or inspected, embedded, surface-conformable eddy-current coils could be favorably attached to the surface of a structural component during its initial assembly or maintenance overhaul. The coils can be manufactured in thin configurations, printed according to the geometry of the part or the expected defect type, and be of minimal weight. These can also be largely insensitive to dirt, temperature, and humidity. This versatility makes eddy-current measurement one of the most suitable techniques for in situ condition-based maintenance demonstration. Flexible eddy-current arrays have been proposed for applications where conventional techniques were employed, such as in structural health monitoring [12] and proximity sensing [4,7].

Fatigue cracks originating from fastener holes, especially those starting at the faying surfaces, constitute the source of a large number of structural failures. Spiral coils, centered on fastener holes, represent a suitable geometrical option for detecting radial cracks. The coils could be designed with high inductance, low resistive losses, and low power requirements. In addition, when coils are manufactured as light, thin, flexible, and surface-conformable printed electronics, they are suitable for integration in challenging applications and could fulfill the geometrical and sensing requirements. However, issues with transducer degradation, durability, and reliability need to be carefully investigated before large-scale deployment. In comparison, surface-attached piezoelectric sensors for ultrasonic inspections are known to have coupling challenges and are sensitive to environmental temperature, humidity, and structural strain [5,7,13]. The PCB coils on flexible polyamide substrates could be designed in different geometries and configurations in order to meet specific structural-monitoring needs [12].

Previously, the attached flexible PCB coils proved to be sensitive to electrical discharge machining (EDM) notches in static conditions [1]. The study documented in this article is a step toward more realistic applications: fatigue cracks developed from starter notches radially introduced around circular holes in aluminum alloy coupons under the condition of applied tensile load, and it has applications in the aviation industry [8,17].

## 2. Materials and Methods

The voltage induced in a sensing eddy-current coil is directly proportional to the rate of change of the current flowing through it. The proportionality factor is the inductance of the coil. This depends on the geometry, electrical conductivity, and magnetic permeability of the test piece [18]. It is important to note that in addition to the coil’s self-inductance, there is also the mutual inductance between the coil circuit and the equivalent circuit of the eddy currents in the test piece. The mutual inductance is opposite in sign to the self-inductance, due to Lenz’ rule [19]. This aspect is included in the mathematical representation of the eddy-current coil impedance, shown below in Equation (1). Therefore, the reactive component of the coil impedance comprises the self-inductance of the coil, L, and the mutual inductance, M, between the coil and the eddy currents induced in the tested part.

(1) Z(ω)=ReZ+j·ImZ=R(ω)+jω·(L−M)

where ω is the angular frequency (ω = 2πf, with f being the frequency), and j is the imaginary unit. In this article, when discussing inductance, the overall effect of self and mutual inductances is considered. Changes in the coil impedance, inductance, resistance, current or voltage amplitude, phase, or other parameters could be used to detect material discontinuities.

**Figure 1:**
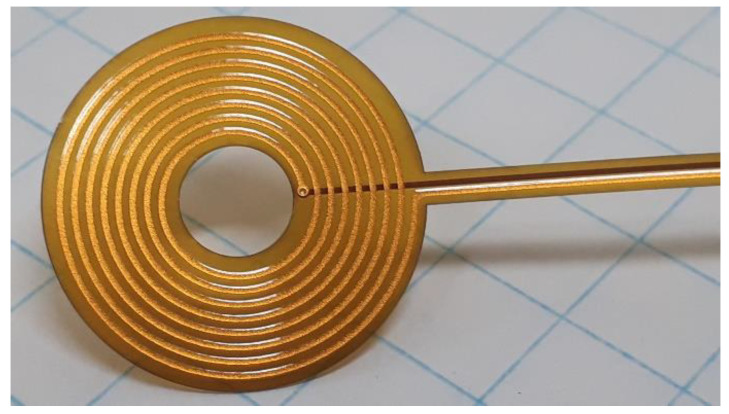
Picture of a PCB spiral copper coil.

In Figure 1, a spiral copper coil, similar to those used in this work, is shown. For estimation of the coil size, it is worth mentioning that the background squares had sides of 5 mm. In this case, the coil was printed by etching on a polyimide substrate.

In addition to material properties, the electrical parameters of a spiral coil depend on its geometry: shape and size, trace width, thickness, and spacing [19,20,21]. The geometry dictates the coil range of operating frequencies and sensitivity [9,22]. Similarly to power transmission design, higher inductance is desired [23]. According to the literature [22,24], the inductance of a spiral coil of outer diameter d_out_ and inner diameter d_in_, with n turns, is given by Equation (2).

(2) $$L=\frac{\mu \cdot n^{2}\cdot d_{\mathrm{avg}}}{2}\left[\ln\left(\frac{2.46}{\rho }\right)+0.20\cdot \rho^{2}\right]$$

where μ is the permeability of the core, d_avg_ is the average diameter of the coil, and ρ = (d_out_ − d_in_)/(d_out_ + d_in_) is the coil’s fill factor. In the present study, the coil has seven turns (n = 7), the inner diameter of the coil is approximately 5.70 mm, and the outer one is 13.5 mm. The coils were 17.5 μm thick printed-circuit-board copper traces on polyimide substrate—50 μm thick. Another layer of polyimide was laid on top of the copper coil, for insulation purposes. With both trace width and inter-trace spacing of 300 μm, the inductance was calculated with Equation (2) to be 542 nH. The maximum current rating for such a cross-section of the copper trace is 0.8 A at 20 °C.

Although the eddy-current technique could not be generally applied to inspect composite structures, it could be used to inspect metallic substrates through composite materials. When in proximity of electrically conductive materials, the magnetic field follows an exponential decay with depth from the surface. As such, the standard depth of penetration (or skin depth), δ, is defined as the depth in the conductive material at which the magnetic field decreases to 37% of its value at the surface and is given by the following relationship:

(3) $$\delta =\frac{1}{\sqrt{\pi \cdot \mu \cdot \sigma \cdot f}}$$

where μ and σ are the magnetic permeability and electric conductivity of the material, and f is the excitation (driving) frequency. A high frequency reduces the field penetrability but increases the eddy-current density close to the part’s surface, making it more sensitive to surface cracks.

For a 7075-T6 aluminum alloy of 31% IACS (International Annealed Copper Standard) conductivity (18.59 MS/m meaning a conductivity of 31% of that of pure copper, i.e., 59.98 MS/m), the skin depth values, calculated for the empirically employed frequencies, are displayed in the Table 1.

As the monitored fatigue crack in this study extended through the entire thickness of the specimen, the depth of penetration indicates the degree of interaction of the eddy current with the discontinuity presented by the crack. However, the reader should note that higher frequencies are more susceptible to noise due to surface irregularities, such as scratches.

### 2.1. Experimental Design

Structurally attached or embedded PCB eddy-current sensors need to be electrically insulated from the metallic parts they monitor. This is achieved using a thin non-conductive substrate on which the coil is printed, such as a polyimide or Kapton^®^ film, avoiding electrical contact and reducing the possibilities of galvanic corrosion. The coil traces are normally encapsulated between polyimide film layers approximately in 50 μm thickness, with only the ends of the connectors exposed. The stationary eddy-current planar coil is meant to provide a localized inspection within its footprint, and when installed around the fastener hole, it provides an excellent inspection opportunity for the detection of radial fatigue cracks initiating from the bore. Fatigue cracks from the bolt-hole propagate along the radial direction and are perpendicular to the surface of the hole [11]. In this study, the spiral coils were operating in absolute mode, meaning that the same coil was used for both excitation and sensing. The excitation current through the coil produces a magnetic field that induces eddy currents in the test part. The secondary field produced by the eddy currents combines with the primary one and modifies the voltage drop across the coil.

The parameters of the fatigue crack-monitoring coil could be compared to those obtained at the beginning of the test or against the parameters of a reference coil. The first approach is rigorous in terms of maintaining the same area of the part under investigation during the entire fatigue test. Comparing against a reference coil, although it might have minor variations in local properties of the part or positioning of the coil, has the advantage of examining against a coil undergoing the same empirical conditions as the one in which we monitoring are the crack. Moreover, this could be performed using the same auxiliary instrumentation for both coils that is available at the time of the test. Depending on the testing situation, one type of referencing may provide more sensitive results than the other.

**Figure 2:**
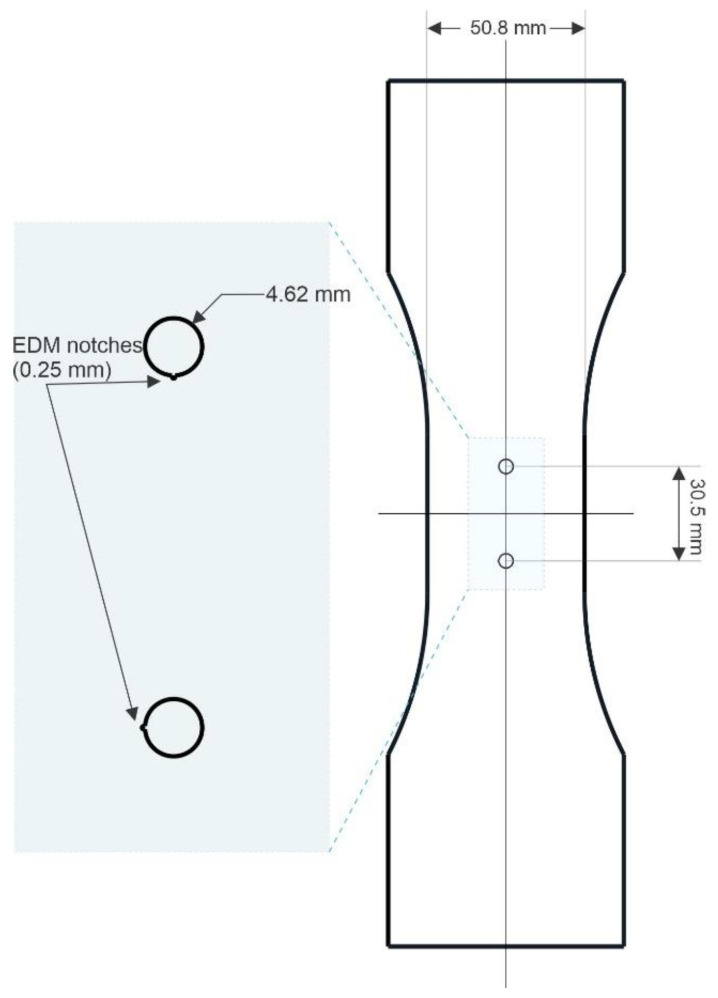
Schematic of the fatigue test coupon, detailing the monitored holes and the EDM notches.

Test coupons of 7075-T6 aluminum alloy, of 0.25” (6.35 mm) thickness and with a gauge area of 2” × 2” (50.8 × 50.8 mm) containing two holes 0.182” (4.62 mm) in diameter placed at a center-to-center distance of 1.2” (30.5 mm) were used. A drawing of the specimen is displayed in Figure 2. The holes contained starter EDM notches of 0.010” (0.25 mm) wide (circumferentially) and 0.010” (0.25 mm) deep (radially). One of the two notches was oriented parallel to the loading direction, whereas the other notch was oriented perpendicular to the loading axis. The role of the notches was to predetermine the location and direction of the fatigue crack nucleation and growth. The notch oriented parallel to the loading axis was introduced for referencing purposes, as no crack was expected to grow from it. The notch oriented perpendicular to the loading axis acted as a stress concentrator to favor local plastic deformations and crack growth. This was done to promote crack nucleation and to monitor crack growth using the second attached coil. The orientation of the EDM notches is illustrated in the zoomed-in view of Figure 2. Therefore, at the beginning of the test, both coils (the reference one and the crack monitoring one) were interrogating the same geometry: a hole and a radial notch 0.010” (0.25 mm) in length. The test piece was cyclically loaded at 15 Hz using a sine wave with an amplitude of 8 ksi (55 MPa or 4000 lbf). The maximum load represents approximately 12% of the material yield strength. The stress ratio, R_σ_, was selected to have a value of 0.1.

**Figure 3:**
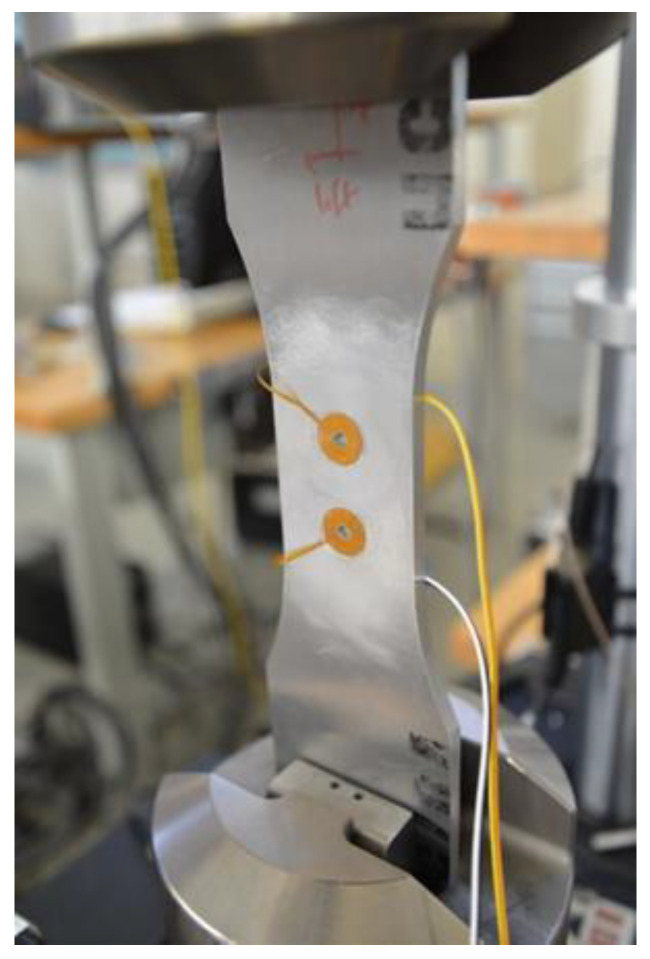
Photograph of the test coupon; the fatigue crack was expected to grow from the lower hole.

The specimen was mounted vertically in a loading frame and clamped using hydraulically actuated grips, as shown in the following photograph (Figure 3). Initially a non-instrumented coupon was used to verify the crack growth duration and determine the viability of using marker bands. The other coupon was instrumented with the two surface-bonded coils, as shown in Figure 3. Alignment of the sensor sensitivity direction with the crack propagation and proper bonding to the test piece are factors that affect the efficiency of the in situ crack monitoring [14]. Selection of the bonding material needed careful consideration, as a rigid bond would transfer load to the sensors and cause them to fail during the crack growth, whereas a weak bond could cause the sensors to de-bond during loading [16].

A flexible, thin, and strong bond is necessary for the successful use of structure-attached monitoring sensors. For bonding of the coils to the coupon’s surface, a commercially available adhesive SI 5140RTV from Loctite [25] was used. Its elongation at break is indicated to be 150% [25]. This silicone-based adhesive had a thickness of approximately 100 μm, and it was expected to reduce the load transfer to the coil. The probing magnetic fields rapidly decrease with distance, but placing the sensor in contact with the surface to monitor and using a differential probe will increase the discontinuity-detection capabilities [26]. Having a localized receiver and a probe larger than the target discontinuity decreases the resolution and sensitivity of the technique, and the differential approach is meant exactly to ameliorate these shortcomings [26].

**Figure 4:**
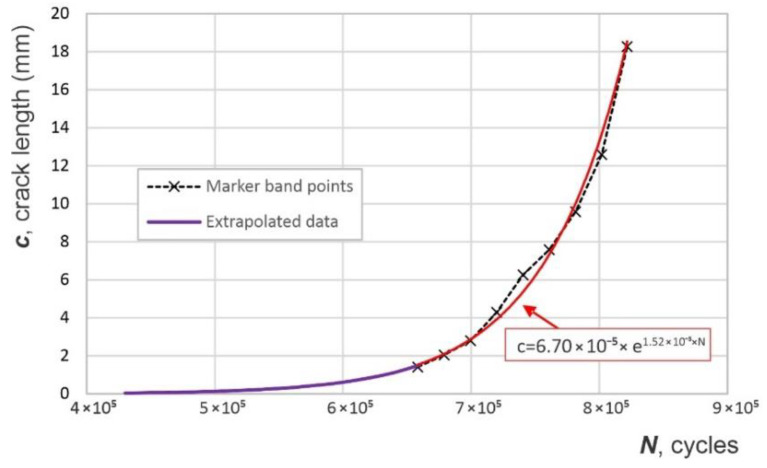
Crack growth measured using marker bands; crack length, c, as a function of number of cycles, N.

In order to determine the crack growth curve, a non-instrumented coupon was used for quantitative fractography using marker bands [27]. These marker bands are groups of striations that, when microscopically analyzed on the crack’s fracture surface by optical means or by scanning electron microscope, determine the locations of the markers in relation to the number of cycles applied, i.e., the advancing fatigue crack. Series of 500 cycles at 15 Hz, with loads between 85% and 60% or between 85% and 10% of the maximum load of 8 ksi, were used to produce “bar-coded” markings. Based on quantitative fractography, the crack size as a function of the number of cycles is presented in Figure 4. The first marker band was found at 1.412 mm from the EDM notch tip. Periodic marker bands were introduced until the end of the test, and the data interpolation revealed an exponential growth pattern. The data was back-extrapolated to estimate the crack-growth curve for shorter cracks than 1.412 mm.

Based on the evaluation of the marker bands of crack growth data and the fitted crack growth model presented in Equation (4), it was found that approximately 350,000 cycles were required to grow the crack from 50 μm, the assumed nucleation length, to 10 mm in length.

(4) $$c={6.70\;\times \;10}^{-5}{\;\times \;e}^{{1.52\;\times \;10}^{-5}\;\times \;N}$$

where c represents the crack length in mm and N is the number of cycles.

The two spiral coils shown in Figure 3 and a current-limiting resistance were connected in series. While the top coil is used for referencing purposes, the bottom coil was monitoring the growing fatigue crack. The voltage drop across the current-limiting resistance was used to determine the current through the circuit. The loading sequence used for the non-instrumented 7075-T6 aluminum alloy coupon was repeated for the instrumented one. The fatigue test was stopped at specific cycling intervals, and the voltage across the two coils was recorded for a set of different driving frequencies.

### 2.2. Numerical Evaluation

Numerical modeling plays an important role in coil design, inspection optimization, and data interpretation. Analytical descriptions of diffusion-based phenomena, as in the case of eddy currents, are complex and approximate. Changes in the coil’s electrical parameters, such as impedance, resistance, inductance, and output voltage (amplitude and phase), are normally used to discriminate test piece properties related to lift-off, thickness, electrical conductivity, and magnetic permeability. Numerical models are employed not only to optimize the coil geometry and parameters for an effective electromagnetic interaction between the sensor and the part, but also to understand and interpret the experimental results [1,28,29].

In this work, physics-based finite element software was employed to simulate interactions of the spiral coils with materials of different conductivities. Various parameters could be evaluated to indicate the best features to analyze. Finite element modeling software, Comsol Multiphysics, version 6.0, and its AC/DC module [30] with Magnetic Fields and Electrical Circuit physics, were employed. Two-dimensional (2D) simulation of the spiral coil of the geometry analyzed the effects of the driving frequency and specimen electrical conductivity on the impedance changes. The Magnetic Fields interface was used to calculate and visualize magnetic fields and induced current distributions around coils and conductors. The interface solves Maxwell’s equations formulated on magnetic vector potential. Additionally, the Electrical Circuit interface was employed by the AC/DC module of Comsol Multiphysics. This allows the introduction of current and voltage sources; and resistive, inductive, and capacitive devices [30]. The two-dimensional and axially symmetric (i.e., 2D axisymmetric) geometries were chosen as appropriate representations of the experimental test conditions. These models also present the advantage of fast running times, since they are not computationally intensive.

**Figure 5:**
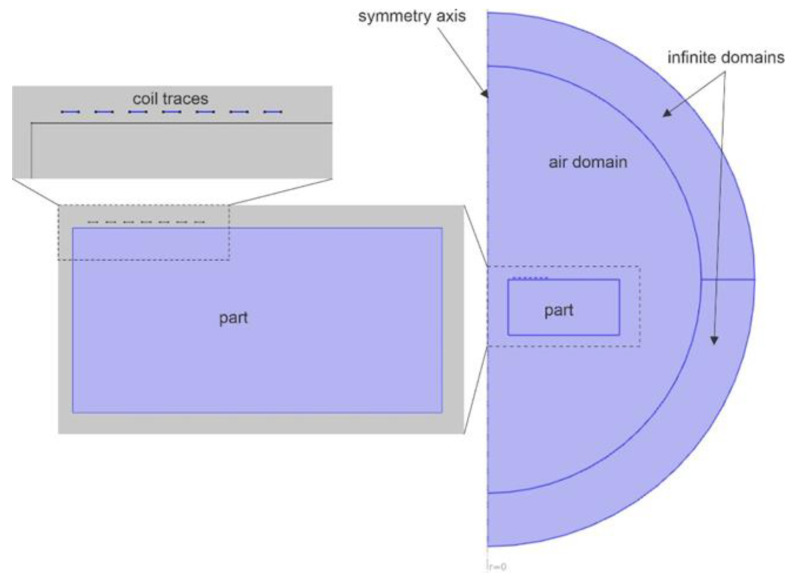
Geometry of the simulated 2D axisymmetric problem representing the part and the coil.

Although a spiral coil does not have any true symmetry, in a first instance, the coil’s excitation and output parameters were analyzed in Comsol Multiphysics, AC/DC Module, Magnetic Fields interface, frequency domain studies in a 2D axisymmetric geometry representing a series of concentric ring traces. As a representation of the empirical studies, a 7-turn planar coil, with a trace 300 μm in width and with a 300 μm inter-trace gap, at a lift-off of 0.2 mm, was modelled as placed atop of a metallic disc of 14.8 mm radius and 6.25 mm height, containing a hole with a 2.31 mm radius. From a modeling perspective, this represented an identical geometric and electromagnetic replication of the experimental case. Due to the small coil thickness, the copper trace was modelled as a boundary in order to avoid potential issues with very fine two-dimensional mesh (as when the coil is modelled as a two-dimensional model). A semi-circular air domain with a radius of 30 mm is placed around the geometry of interest. A semi-circular layer of 6 mm width is used for defining infinite domains. These are artificial domains which are used to provide virtual domain scaling, to stretch the finite element domains in the radial direction in such a way that the physics of boundary-domain conditions are effectively satisfied at the outer layer of the infinite domains. The magnetic insulation at the outer-domain boundaries essentially represents a boundary where the tangential component of the magnetic vector potential and the normal component of the magnetic field are zero. The sensitivity of planar coils increases as they are driven at frequencies approaching resonance, which are typically in the region of tens or hundreds of MHz [9,31]. In this study, a constant-amplitude current of 100 mA drove the coil at various frequencies in a range from 10 kHz to 10 MHz. The axisymmetric geometry analyzed at this step is shown in Figure 5. In this instance, the current-driven coil was employed to ensure a constant magnitude magnetic flux coupled with the part.

**Figure 6:**
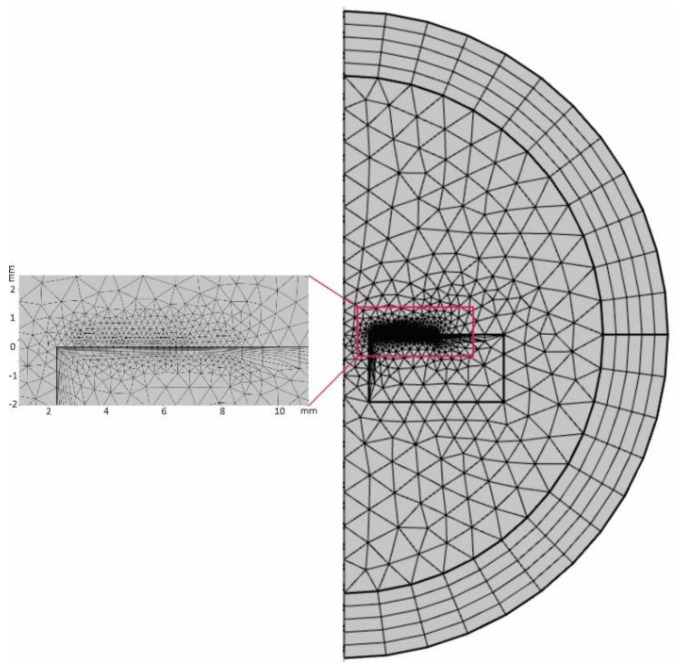
Visualization of the meshed geometry for the coil, part, and infinite domains.

The complete mesh contained approximately 2000 domains and 150 boundary elements. The maximum element size of the boundary domain represented by the coil trace was half of its width, i.e., 150 μm. Moreover, the side of the part facing the coil was meshed using closely spaced twelve boundary layers to better represent the region of interest. The mesh of the geometry is shown in Figure 6.

## 3. Results

This section presents the results of the modelling evaluations, their implementation in the laboratory experiments, and the empirical observations and analysis.

### 3.1. Simulation Results

**Figure 7:**
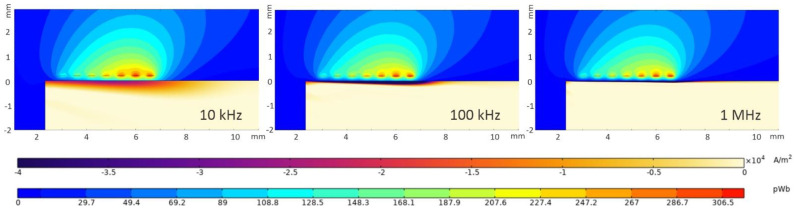
Magnetic flux in the air domain and current-density distribution in the part at coil driving frequencies of: 10 kHz, 100 kHz, and 1 MHz.

The magnetic flux in the surrounding air domain and around the coil traces, and the eddy-current density in a specimen of conductivity of 31% IACS for a current of 100 mA through the coil, at excitation frequencies of 10 kHz, 100 kHz, and 1 MHz, are shown in Figure 7. The coil was considered at a distance of 0.2 mm from the part. For comparison purposes, the color scales for the three frequencies are identical. From these plots, it can be observed that the magnetic flux in air did not change significantly with the driving frequency, as the driving current was constant. On the other hand, the induced eddy currents in the part indicate a lower depth of penetration and higher intensity with increasing frequency. The eddy current’s amplitude increases, and its distribution in the material decreases, with increasing frequency.

An important aside needs to be mentioned here to indicate the fact that a surface breaking discontinuity, as a fatigue crack, is essentially a barrier in the free flow of the induced eddy currents, and it is equivalent to a local drop in material conductivity (or an increase in resistivity). In eddy-current testing, changes in the electrical parameters of the sensing coils are used to characterize the material under inspection. The resistance of the coil, its induction, and its impedance were determined based on the current-driven model discussed above. The coil overall inductance has an almost constant value while the coil is in air, i.e., 540 nH, as this contains only the self-inductance component and no mutual inductance component (see Equation (1)). The value of the inductance with the coil in air was close to the 542 nH value obtained using Equation (2). This is because the self-inductance of the coil is independent of the current flowing through it, but the mutual inductance changes when the coil is placed near a conductive object. The effect of the eddy currents is most visible at low electrical conductivities of the part and low driving frequencies, as these parameters allow deeper penetration and a more pronounced effect of the mutual inductance due to the flow of induced currents.

**Figure 8:**
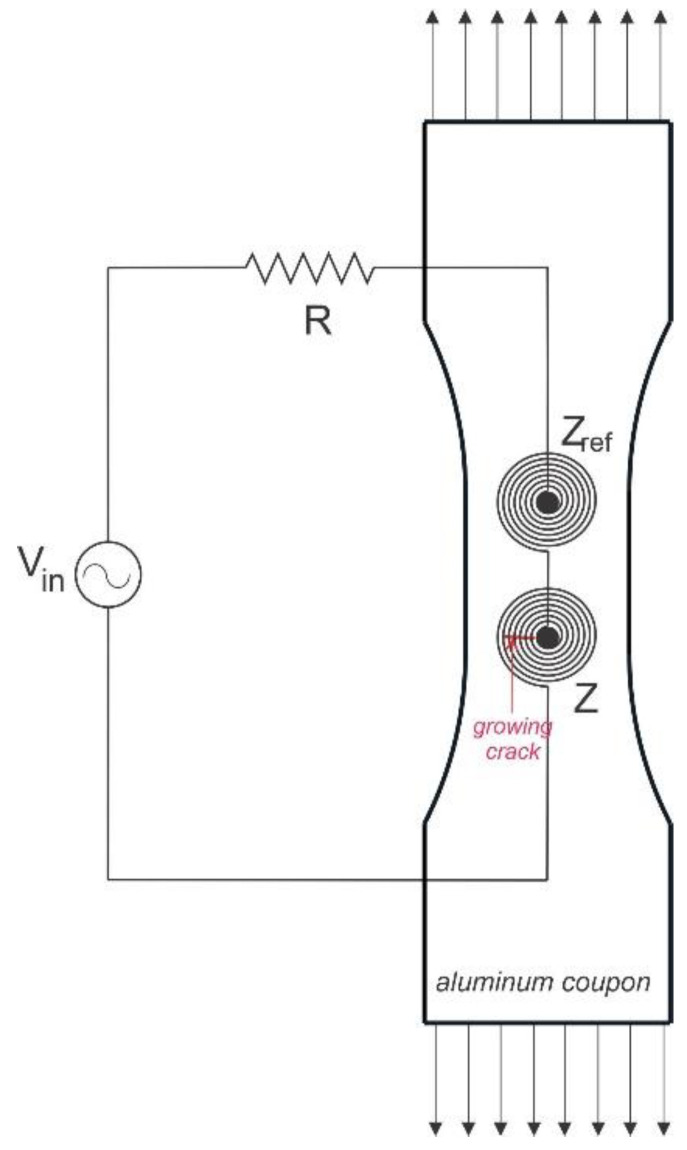
Schematic showing the measuring circuit with the coils attached to the coupon under tensile loading.

From a practical implementation point of view, the coil impedance could be used as the monitoring feature of the specimen under test. The indirect measurement of changes in the coil impedance, as based on measurement of the voltage drop across it and the current flowing through it, is simple to put in practice. However, even in this case, the changes could be minute and difficult to identify with standard laboratory instrumentation, and differential measurement against a reference specimen is necessary. For this purpose, as described in the experimental design section, one of the coils in the coupon was used as a reference for monitoring the crack growth. The two coils were identical, and they generated the same magnetic field, due to being connected in series and to an AC voltage source of 2 V. The testing circuit is schematically shown in Figure 8. The current through the circuit was measured based on the voltage drop across a current limiting resistor of 1.15 Ω.

**Figure 9:**
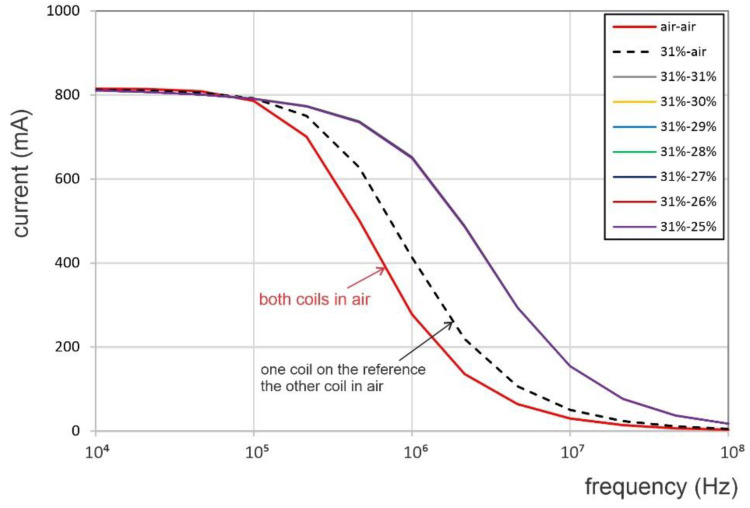
The value of the circuit current with frequency when the coils are either in air or on parts of different conductivities (curves are overlapping for different conductivity pairs).

A conductivity drop of at least 3% IACS was reported to be due to the presence of a fatigue crack [12]. The current through the series circuit shown in Figure 8 changes with the electrical load. Its variation with frequency and for different material conductivities is plotted in Figure 9. In the first two instances, both coils are considered to be in air, and then, one coil is placed on the reference and the other is kept in the air. In all other cases, the reference coil was considered placed on a part with a fixed conductivity of 31% IACS, similar to that of the aluminum alloy used in the experimental study, and the second coil was placed on materials with progressively lower conductivities, from 31% to 25% IACS in decrements of 1% IACS. The lower conductivity for the hole monitored by the measuring coil was meant to simulate a growing crack, as this is an impediment to flowing eddy currents. Although this is a rough approximation, it identified the trend in the measurement parameters of the coils. The results shown in Figure 9 indicate a very small change in the circuit current; the current curves are overlapping for when both coils were placed on electrically conductive specimens, regardless of their conductivity differences. As a consequence, this enforced the same excitation magnetic field.

**Figure 10:**
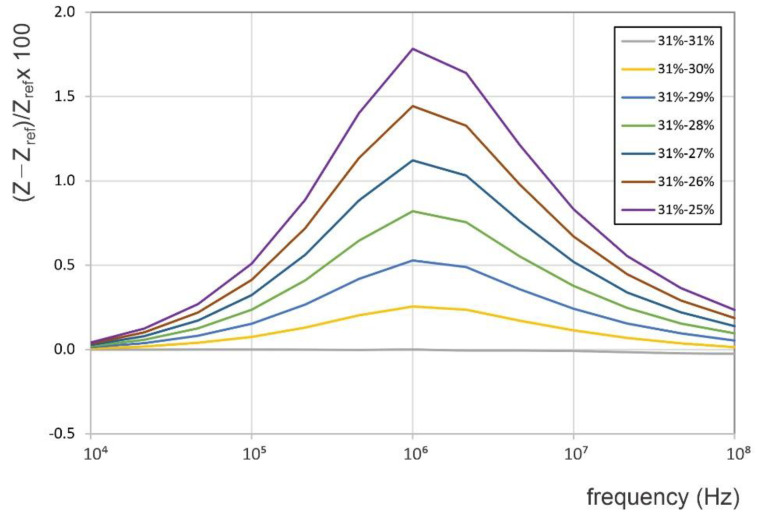
The relative change in coil impedance with frequency for different conductivity pairs.

In practical situations, the electrical parameters of the coil placed in air could not be reliably replicated at the test time. For this reason, it is customary to have a reference specimen with known characteristics that the measuring coil parameters could be compared against. In this case, the reference coil was placed on a part with conductivity of 31% IACS, and the measuring coil was considered on a part of electrical conductivity ranging between 25% IACS and 31% IACS. The relative change in the coil impedance with the driving frequency varying from 100 kHz to 10 MHz is shown in Figure 10, where Z and Z_ref_ denote the coil impedance when it is placed on a material of changing conductivity or on the reference, respectively.

**Figure 11:**
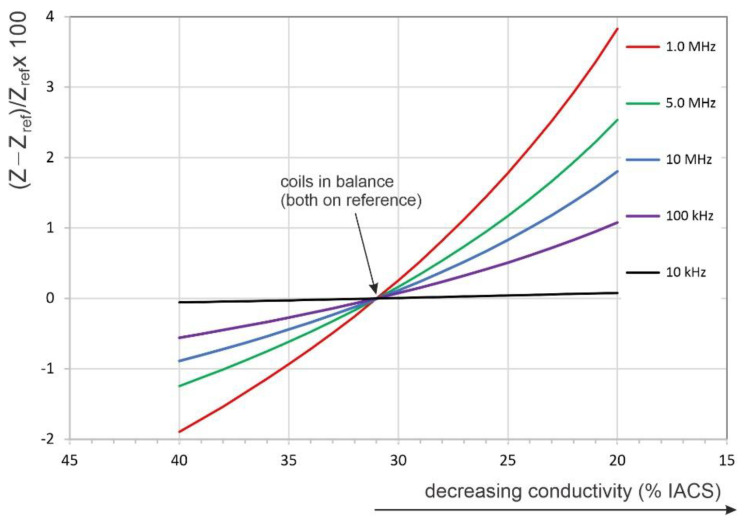
The relative change in coil impedance for discrete frequencies and continuous variation in conductivity.

As seen in Figure 10, the highest relative change in the electrical impedance was around 1 MHz driving frequency, regardless of the conductivity pairs considered. The changes in the relative impedance with respect to the reference value (i.e., 31% IACS) on the same geometry, but with decreasing electrical conductivity in the range from 40 to 20% IACS, are shown in Figure 11, for different driving frequencies. This result suggests that the relative impedance increases monotonously with decreasing conductivity. However, as can be observed from Figure 11 (and inferred from Figure 10), the sensitivity of the relative impedance to changes in conductivity increased from 10 kHz to 1.0 MHz, after which it decreased for higher frequencies. Based on these numerical results and the feasibility of empirical measurements, it was concluded that the change in the coil impedance, i.e., the relative impedance change in the crack-monitoring coil with respect to an identical coil placed on a reference location, is a sensitive feature for observation of a growing crack.

### 3.2. Experimental Results

Flexible PCB coils could be manufactured at low cost and minimal weight to have adequate inductance and low power consumption, but also to have the optimal geometry for the specific discontinuity type, size, and orientation sought. For example, the distance between the probe and the test piece, called lift-off, affects the mutual coupling between the two. A small lift-off value increases the electromagnetic interaction between the part and the transducer. While there are ways to minimize its effect (i.e., as lift-off compensation phase adjustments on an impedance plane diagram representation), for embedded, surface-conformable eddy-current coils, the lift-off effect is constant. In this case, the lift-off consists of the layer of protective polyimide of the copper trace and the thin layer of adhesive used to attach them to the metal structure. Although the adhesive layer’s thickness could easily be kept constant, its elastic properties play an important role in transferring the load from the structure to the sensor itself.

The cycling intervals went from 82,000 cycles at the beginning of the test to 20,000 cycles in the later stages of the test, when the crack growth was expected. Based on the marker bands estimation, determined using the non-instrumented specimen, no fatigue crack growth was expected prior to 500,000 cycles. The test was stopped when a fatigue crack was visible with a magnifying glass and in conditions of static tensile loading at 8 ksi, between 20,000 cycle intervals. For the instrumented specimen, the test was stopped after 1025,000 cycles.

**Figure 12:**
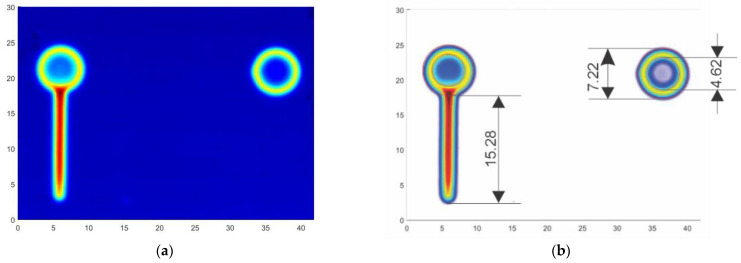
Estimation of crack length at the end of the fatigue test: (**a**) raw data, (**b**) processed data for crack length evaluation.

Since crack growth does not take into account the number of cycles necessary to nucleate the crack, and this could depend on factors out of empirical control, the curve determined via marker bands (Figure 4) is assumed to be represented by the same equation but with different nucleation times. Consequently, this means to start from a certain crack length and corresponding number of cycles at the end of the fatigue test and determine when the crack started growing from the crack growth curve. A non-destructive way to evaluate the crack length is using visual measurements while the specimen is statically loaded and the crack is open. However, even in this case, the crack length measurement could be affected by large uncertainties. A more accurate approach to measure the crack length is to perform surface eddy-current scans after the specimens are removed from the load frame. Eddy current results were obtained by surface scanning of the backs of the specimens (i.e., the side opposite to the one on which the coils were attached) using an absolute pencil probe at 50 kHz driving frequency and a scanning increment of 0.2 mm. The scanned surface had dimensions of 30 × 42 mm, and the raw scans are shown in Figure 12a below. The coordinate dimensions are given in mm. For accurate sizing of the fatigue crack, the diameter of 4.62 mm of the reference hole and the eddy-current technique over-estimation of the crack, as illustrated in Figure 12b, were considered. Based on these estimations, it was found that the crack was approximately 15.28 mm long at the end of the fatigue test. When the starter notch length of 0.25 mm was taken into account and subtracted from this estimation, it was concluded that the actual fatigue crack was 15.0 mm long. The fatigue crack was assumed to have a rectangular shape and penetrate through the full thickness of the coupon.

**Figure 13:**
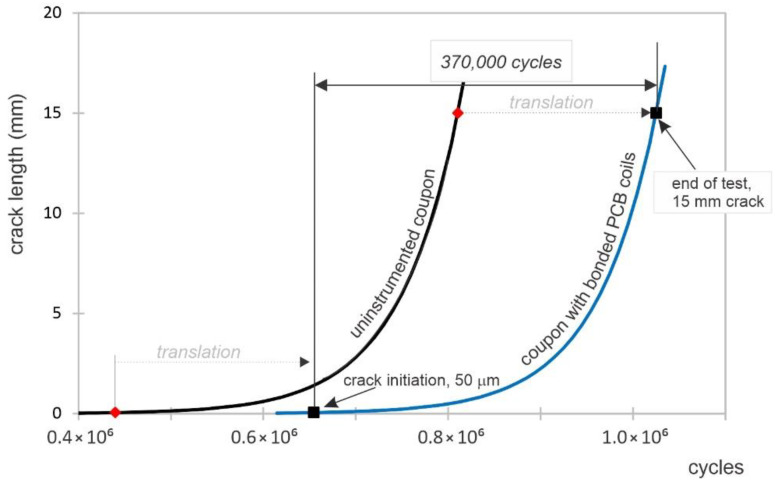
Estimation of number of cycles from crack initiation to the end of the test.

The crack nucleation is dependent on many macroscopic and microscopic variables. While the bulk properties and loading parameters for the two specimens used in this study are similar, the microscopic non-homogeneities are determining factors that affect when the crack starts growing. The crack on the instrumented coupon was assumed to have the same growth curve as the one on the non-instrumented coupon, but with a different crack initiation point, as shown in Figure 13. We found that there were about 370,000 cycles between the crack initiation, i.e., ~50 μm, to a crack of 15.0 mm, the crack length at the end of the test.

As shown previously, the electromagnetic simulations indicated that the relative change in the coil impedance Z with respect to that of the reference coil Z_ref_ is sensitive to changes in the electrical conductivity of the part. The presence of a crack locally lowers the electrical conductivity, and this feature was proposed for crack monitoring. Based on simulation results and empirical observations, frequencies around 1 MHz were expected to show better crack length sensitivities, and these cases were further analyzed.

**Figure 14:**
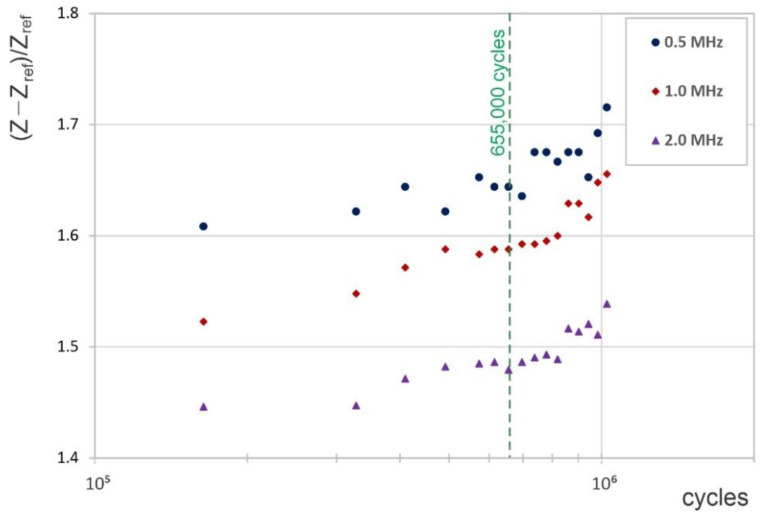
The relative change in impedance with the number of cycles, as experimentally measured.

The fatigue test was stopped after 1025 k cycles for the instrumented specimen. The surface-eddy-current and marker-bands crack-growth analyses indicated that the crack initiated at about 370,000 cycles prior to the end of the test, meaning that the crack started growing after about 655,000 cycles from the beginning of the fatigue test, as indicated in Figure 14. Figure 14 shows the effect of the driving frequency on the relative impedance for 0.5, 1.0, and 2.0 MHz. As observed, the relative impedance started to increase prior to the estimated crack initiation point. This might have been due to the measuring coil being sensitive to local changes in the material properties, such as localized conductivity variations, prior to actual crack initiation.

**Figure 15:**
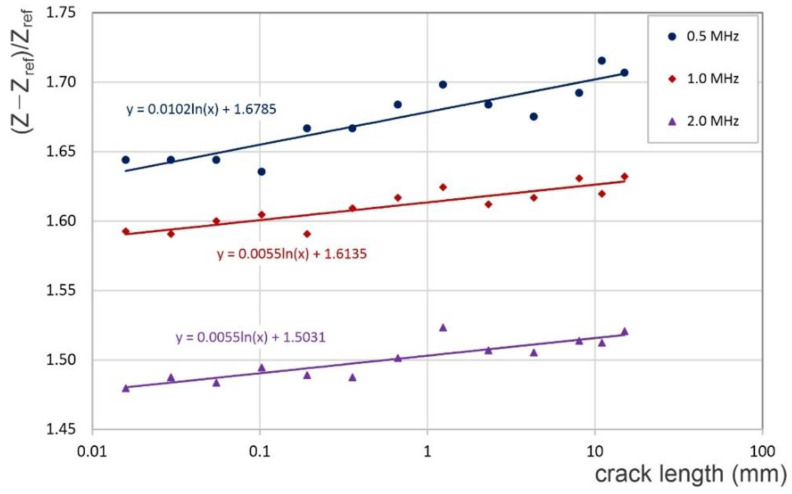
The experimental relative change in impedance with the crack length for three driving frequencies.

Using the translated crack growth curve from Figure 13, the relative change in the coil’s impedance is shown with respect to the crack size in Figure 15. The semi-logarithmic graphic representation displays the relative impedance variations with the crack length. These have a linear behavior, on the semi-log scale, with the crack length all the way to the end of the test, i.e., at a crack length of 15.0 mm. As seen in Figure 15, a lower driving frequency (0.5 MHz) resulted in better sensitivity to crack length than higher frequencies. However, it was observed that data scatter increases with decreasing frequency, and consequently, the optimal test procedure should be selected by balancing sensitivity and scatter. Although the two coils were not perfectly balanced in the experimental investigation, the behavior of the relative impedance change was similar to that found through the numerical simulations and displayed in Figure 11.

## 4. Discussion

Structural health monitoring applied for strain, vibration, and temperature measurements (strain gauges, accelerometers, thermocouples) are not meant to detect damage. On the other hand, SHM sensors for damage detection have the role of in situ non-destructive inspections (as for diagnosis) and on-line monitoring for preventive measures (as for prognosis). Condition-based monitoring is a required component of predictive maintenance and has the potential to increase the availability and safety of metallic structures while reducing costs associated with periodic inspections. Additionally, it has to be considered for parts and components not designed for inspectability.

The present work aims at the detection of cracks open to the surface on which the eddy-current coil is bonded, either a through-wall or a partial penetration crack. When a partially wall-penetrating crack is open at the far side (i.e., opposite to the coil side) of the part, the driving frequency may be adjusted to assure sensitivity of the approach at the depth in the material at which the crack is located.

The development of eddy-current condition assessment and monitoring with embedded sensors is still in its incipient stages, but the results obtained so far are showing potential for condition-based assessment of metallic aircraft structures. The laboratory and numerical studies considered detecting and monitoring a growing crack in dynamic conditions. Low cost, custom geometry, surface conformability, flexibility, low weight, suitability for array deployment, and different operating modes, are only a few factors supporting the development of surface-conformable eddy-current transducers. In addition, optimization is required to assure the necessary sensitivity, durability, and reliability of these sensors. Future advancements in this work could also involve evaluating the coil response in a bridge circuit, three-dimensional numerical simulation simulations that take into account the growing crack rather than conductivity changes, and the presence of a fastener and how the fastener material influences the coil response. Cooperation among designers, operators, and maintainers of aircraft structures could facilitate the implementation of embedded sensors not only for diagnostics, but also for prognostics purposes.

Spiral coils with overall thicknesses of less than 100 μm are aimed to be placed in critical areas, such as at the faying surfaces, around the fastener holes, to monitor the growth of radial fatigue cracks. The present study tried to emulate a field test situation. A planar, single-layer winding coil, such as the PCB ones discussed here, will have a small number of turns, resulting in a small inductance, for which minor changes may be difficult to measure. The resistance of the coil varies with the excitation frequency, but measuring the coil impedance against a reference case leaves the coil inductance as the most sensitive feature to a growing crack in the monitored part. The coil’s resistance effect cancels out in this arrangement. A small inductance translates into a high resonance frequency, but a short equivalent length of such a coil has a small resistance as well, allowing for a higher current to pass through (in a voltage driven configuration).

## 5. Conclusions

Printing technologies of copper traces on flexible surfaces are enabling the application of permanently attached or embedded eddy-current probes for condition assessment and monitoring of electrically conductive parts. Their miniaturization, accompanied by small weight and volume, are supporting their widespread application for structural health monitoring. In this laboratory-based empirical study, numerical modeling was employed for sensor design and crack growth monitoring optimization. Electromagnetic simulations were extensively used to guide the experimental analysis for selection of the driving frequency and choosing of the best feature to sense the fatigue crack growth. It was found that a frequency of around 1 MHz would provide the highest sensitivity to fatigue crack length. The relative impedance change in the crack-monitoring coil with respect to an identical coil placed on a reference specimen/location was chosen as the appropriate feature of interest, based on practical applicability and sensitivity to crack growth. At the same time, a flexible bonding material was chosen to increase the coil survivability in the strain field of the crack. Although this work represents a proof-of-concept, the approach is cost effective, and it is simple to implement in practice. However, it requires customization of sensor geometry and parameters, based on the expected discontinuities.

In situ non-destructive evaluation uses sensors to accurately monitor the condition of aircraft structures without causing disruptions to service, necessitating disassembly, or introducing inadvertent damage. The flexible printed-circuit-board eddy-current sensors discussed in this article were designed to be embedded in the multi-layered structures of aircraft, where inspection or monitoring is desired but access and geometry are challenging.

## Figures and Tables

**Table 1:** Standard depth of penetration for 7075-T6 aluminum alloy with various frequencies.

**f (MHz)**	0.5	1.0	2.0	5.0	10
**δ (mm)**	0.165	0.117	0.083	0.052	0.037

## Data Availability

Data generated during this study is not publicly available.
